# Supplementary material for: Essentiality and dynamic expression of the human tRNA pool during viral infection
Source: Mol Syst Biol. 2026 Jan 20;22(4):519–36. doi: 10.1038/s44320-025-00181-7 (PMC13046961; doi:10.1038/s44320-025-00181-7)
Supplement: Supplementary file 1 — Appendix [file 44320_2025_181_MOESM1_ESM.docx]

# Appendix to Essentiality and dynamic expression of the human tRNA pool during viral infection

Noa Aharon-Hefetz, Michal Schwartz, Einav Aharon, Noam Stern-Ginossar, Orna Dahan, and Yitzhak Pilpel

Contents

[Appendix to Essentiality and dynamic expression of the human tRNA pool during viral infection 1](#_Toc216717652)

[1. Appendix Supplementary text 1](#_Toc216717653)

[1.1 Design of a comprehensive sgRNA library for the human tRNA pool 1](#_Toc216717654)

[1.2 Testing the targeting quality of the sgRNA library 3](#_Toc216717655)

[2. Appendix Figure S1 4](#_Toc216717656)

[3. Appendix Figure S2 5](#_Toc216717657)

[4. Appendix Figure S3 6](#_Toc216717658)

[5. Appendix Figure S4 8](#_Toc216717659)

[6. Appendix Figure S5 9](#_Toc216717660)

[7. Appendix Figure S6 11](#_Toc216717661)

[8. Appendix Figure S7 13](#_Toc216717662)

[9. Appendix Figure S8 15](#_Toc216717663)

## 1. Appendix Supplementary text

### 1.1 Design of a comprehensive sgRNA library for the human tRNA pool

In a previous study, we manipulated the human tRNA pool using a CRISPR-based tRNA knockout library targeting several tRNA families to investigate the essentiality of these tRNA families for cell proliferation and cell cycle arrest (Aharon-Hefetz et al., 2020). Here, we aim to expand the scope and generate a new tRNA-CRISPR library to cover the entire repertoire of human tRNA genes, with multiple sgRNAs per tRNA gene. The new CRISPR library we designed comprises sub-libraries in which we targeted both tRNA and protein-coding genes (Fig. 5A). The version of the human tRNA pool we worked with (Chan & Lowe, 2016) consists of 617 tRNA genes covering 49 families of functional cytosolic tRNAs, 54 pseudo-tRNA gene families, and 21 mitochondrial tRNA genes, all of which we have aimed to target here (Fig. 5A). We note that mitochondrial tRNAs are not supposed to be targeted by the current CRISPR/Cas9 editing method since the Cas9 enzyme functions in the cytosol. Thus, at the bare minimum, these tRNAs can be expected to serve as a neutral control for the CRISPR edits, as they are unlikely to be altered. Due to the high similarity between tRNA genes within isoacceptors, i.e., the same anticodon family (with near 100% identity among about half of the tRNA genes belonging to the same isodecoder tRNA family), we could potentially target multiple tRNA isodecoder genes with the same sgRNA. Yet, the sequence similarity between different families may come with the challenge of undesired off-targeting of tRNAs from other tRNA families. To reduce the off-target effect between tRNA families, after we designed sgRNA candidates for each human tRNA gene, we filtered out the sgRNAs that had between 0 to 1 mismatch relative to other tRNAs that were not part of the targeted family, as long as the targeted tRNAs had at least two additional potential sgRNAs. In our final set of sgRNAs, the number of sgRNAs per tRNA family varies significantly between tRNA isoacceptors of both functional and pseudo tRNAs (Appendix Fig. S7A-B). On average, there are seven sgRNAs per functional cytosolic or pseudo tRNA gene, 19.5 sgRNAs per functional cytosolic tRNA family, 17.3 sgRNAs per pseudo tRNA family, and 4.2 sgRNAs per mitochondrial tRNA. In both the functional cytosolic and pseudo tRNAs sub-libraries, the editing position of most of the sgRNAs is downstream of the anticodon (Appendix Fig. S7C-D). The efficiency score of the sgRNAs, based on the sgRNA design algorithm, is highly variable between sgRNAs, yet the distribution of scores is similar for both functional cytosolic and pseudo tRNAs sub-libraries (Appendix Fig. S7E-F). We did not exclude low-efficiency scoring sgRNAs from the final library because some of them were found to reduce the targeted tRNA levels by up to 2-fold in the previous library (Aharon-Hefetz et al., 2020).

Apart from sgRNAs targeting tRNAs, we included in this CRISPR library sgRNAs targeting tRNA-related protein-coding genes (Fig. 5A). Specifically, the changes in certain chemical modifications on tRNAs that we observed during HCMV infection prompted us to test the essentiality of tRNA modification enzymes during HCMV infection. Thus, a sub-library consisting of sgRNAs targeting 84 out of 90 known genes that encode tRNA modification enzymes (Ashburner et al., 2000; Carbon et al., 2009; Consortium et al., 2023) (Fig. 5A) was added. Another sub-library consists of sgRNAs that target other human protein-coding genes that serve as known dependency and restriction factors for the HCMV infection model and cell proliferation, which we call the control group (Fig. 5A). We chose the control genes based on the study of Hein *et al*., where they performed a whole-genome CRISPR library screening in HCMV infection (Hein & Weissman, 2022). We selected between 5 and 8 genes for each subgroup, focusing on restricting and dependent factors for HCMV infection and cell growth (Appendix Fig. S7G). These sgRNAs are thus expected to enhance or reduce infection or cellular growth, respectively. In addition, we added 525 non-targeting sgRNAs (Fig. 5A), which consist of random nucleotide sequences and serve as neutral controls, as is customary in CRISPR-based knockout libraries (Doench et al., 2015). The sequences of sgRNAs we used for the two protein-coding sub-libraries and the non-targeting sgRNAs were taken from the Brunello library (Doench et al., 2015), while the sgRNAs for all human tRNAs were designed in-house. The final design of the construct used to generate the sgRNA library is shown in Appendix Fig. S7H. Our library comprises the first CRISPR sgRNA library designed to target the entire human tRNA pool (see Methods).

### 1.2 Testing the targeting quality of the sgRNA library

We tested the quality of the CRISPR screen experiment by several criteria. In each sample, pairs of sgRNAs that target the same gene are expected to show similar essentiality values compared to teams of sgRNAs that do not target the same genes. Appendix Fig. S8A shows that sgRNAs for the same gene typically behave more similarly than sgRNAs for different genes. Using the same ratio test described above, we compared the performance in our CRISPR screen of our own design of sgRNAs targeting tRNAs, to well-established sgRNAs targeting protein-coding genes (Doench et al., 2015). We found that the targeting quality of our designed sgRNAs against tRNA genes is comparable to the well-defined sgRNAs targeting protein-coding genes (Appendix Fig. S8B). We further tested the representation of the non-targeting sgRNAs in the CRISPR-targeted population. Non-targeting sgRNAs serve as negative controls, and we indeed observe that they are not significantly enriched or depleted in the CRISPR-targeted cells following competition (Appendix Fig. S8C). Next, we examined the quality of the highly ranked sgRNAs, as determined by the MAGeCK tool. We found that high-ranked sgRNAs target tRNA genes with higher expression levels in HFF cells than those targeted by low-ranked sgRNAs (Appendix Fig. S8D). This result suggests that the high-ranked sgRNAs, which contribute to the phenotypic effect, target highly expressed tRNAs within the cellular tRNA pool.

1. Appendix Figure S1


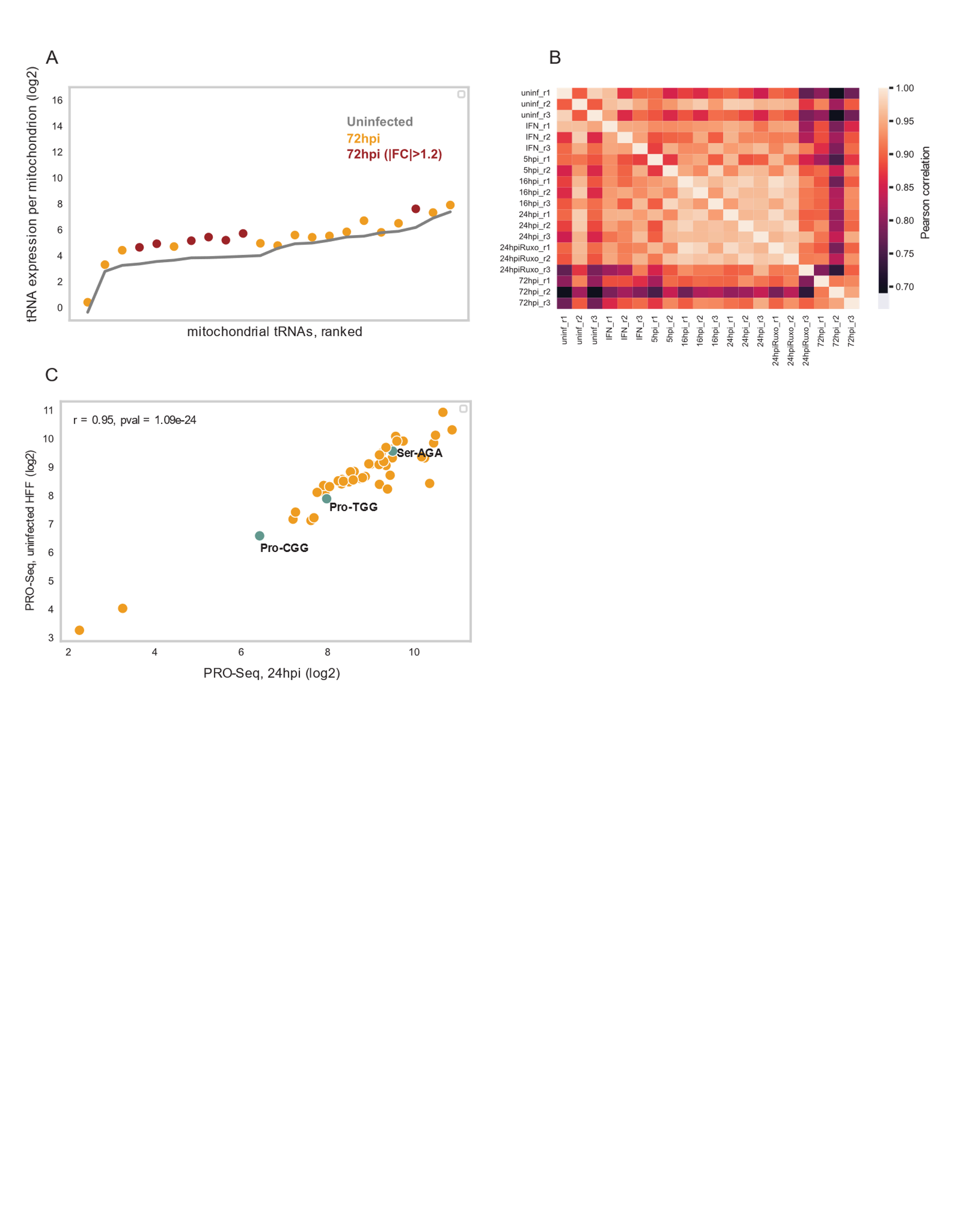


**Appendix Figure S1: Changes in the tRNA levels in HCMV-infected HFF**

A| Mitochondrial tRNA levels (log2) at 72hpi normalized to the typical number of mitochondria per cell (uninfected HFFs -gray line; HCMV-infected cells - orange-red dots). The mitochondrial tRNAs are ordered based on their expression level in uninfected cells. tRNA genes that are differentially expressed (|FC|>1.2) are marked in dark red.

B| Pearson correlation matrix between tRNA levels of all samples and biological repeats.

C| A comparison of nascent tRNA expression levels between HCMV-infected cells at 24hpi (x-axis, log2) and uninfected HFFs (y-axis, log2). The data produced by PRO-SEQ technology was taken from (Ball et al., 2022). Each dot represents the sum expression of all tRNA isodecoders belonging to the same tRNA isoacceptor. The marked tRNA isoacceptors in green refer to the differentially expressed tRNAs that exhibit opposing dynamics between IFN-treated and HCMV-infected cells (Fig. 1F). Pearson correlation: r = 0.95, p-value = 1.09e-24.

1. Appendix Figure S2


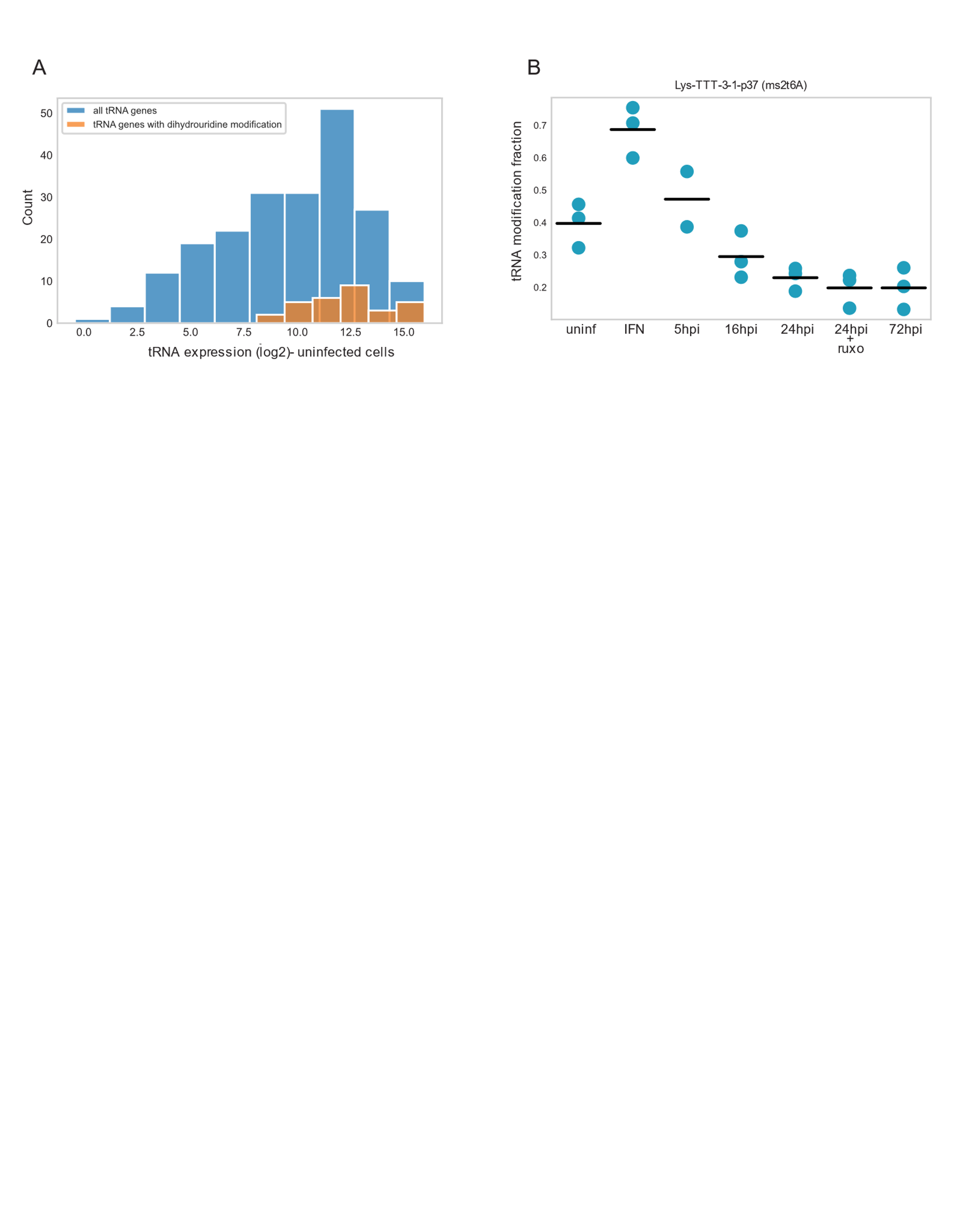


**Appendix Figure S2: Changes in the tRNA modification levels in HCMV-infected HFF**

A| Histograms describing the mean expression level (log2) of all tRNA genes (blue) and tRNA genes carrying dihydrouridine modification (orange) in uninfected cells.

B| Change in the tRNA modification level along HCMV infection on Lys-TTT-5-1 gene, position 37, modification ms2t6A. For each sample, each dot represents a biological replicate (3 replicates in total). The line represents the average modification level in each condition or HCMV infection time point. Shown here is the same modification at the same position and with the same tRNA type as in Fig. 2B, but on a different tRNA isodecoder.

1. Appendix Figure S3


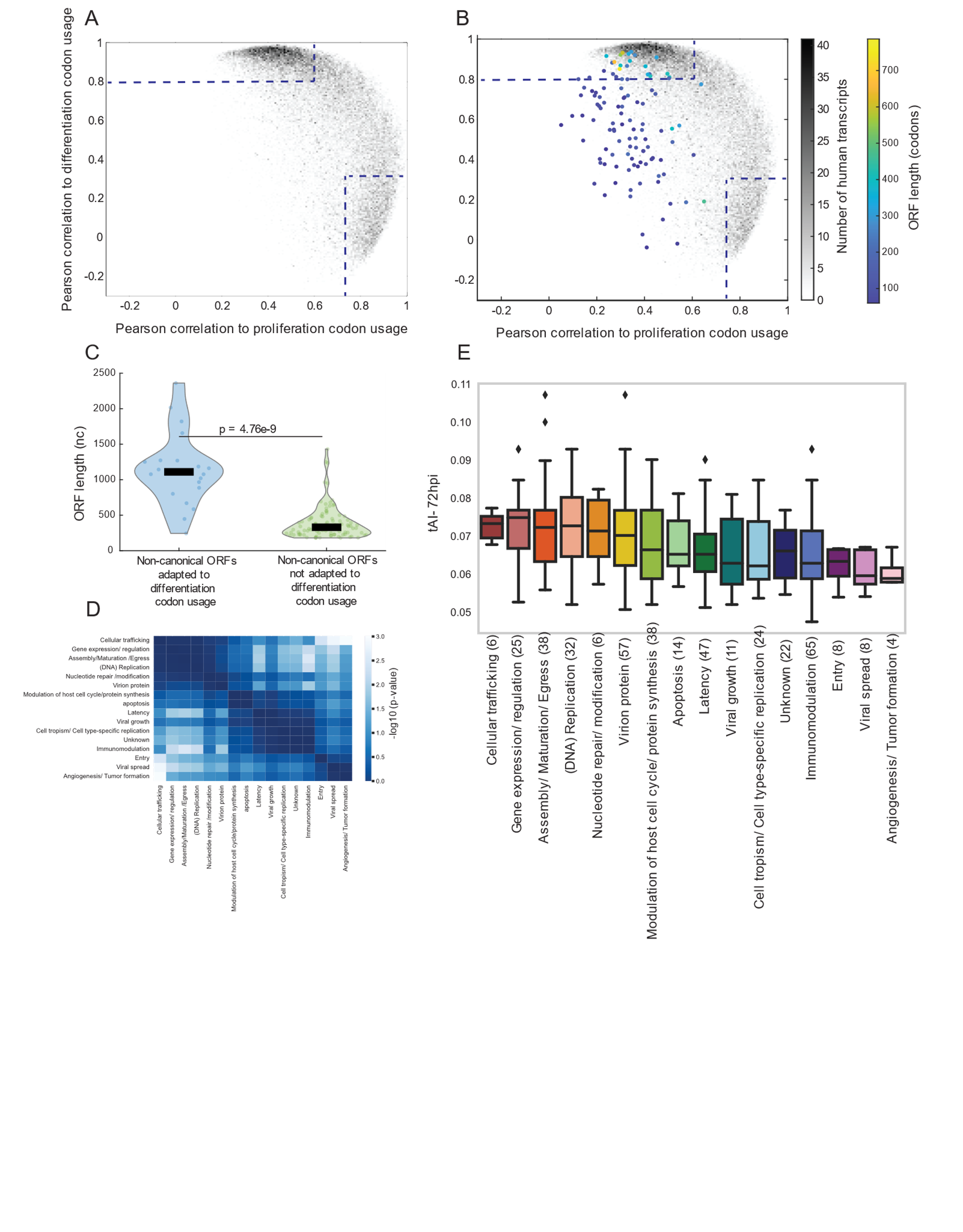


**Appendix Figure S3: Codon usage adaptation of HCMV ORFs to the tRNA pool of HCMV-infected HFF**

A-B| Density plots of the Pearson correlation measured for each transcript to the human proliferation (x-axis) and differentiation (y-axis) codon-usage signatures. Gray dots denote human transcripts. The color bar (gray) represents the number of human transcripts (36762 in total). The dashed lines represent the Pearson coefficients that determine the most prominent groups of human genes with high similarity to the proliferation (18%) or differentiation (30%) codon usage signatures. In B| color dots denote non-canonical HCMV ORFs. The color bar of the non-canonical HCMV ORFs depicts the nucleotide length of the gene. The analysis did not include short non-canonical ORFs (< 58 codons).

C| A violin plot showing the difference in ORF length between non-canonical ORFs that are adapted to the differentiation codon usage (as depicted in Appendix Fig. S3B, r>0.8) and non-canonical ORFs with lower correlation to the differentiation codon usage (r<0.8). Wilcson rank-sum test p-value = 4.76e-9.

D| A heatmap describing the (-log10) p-values of pairwise T-test that test for significant differences in the tAI values between functional gene groups of HCMV.

E| A box plot depicting the tRNA adaptation index (tAI) of canonical HCMV ORFs as calculated based on the tRNA pool of 72hpi. HCMV ORFs are grouped according to their functionality, as was determined by Ye et al., 2020.

1. Appendix Figure S4


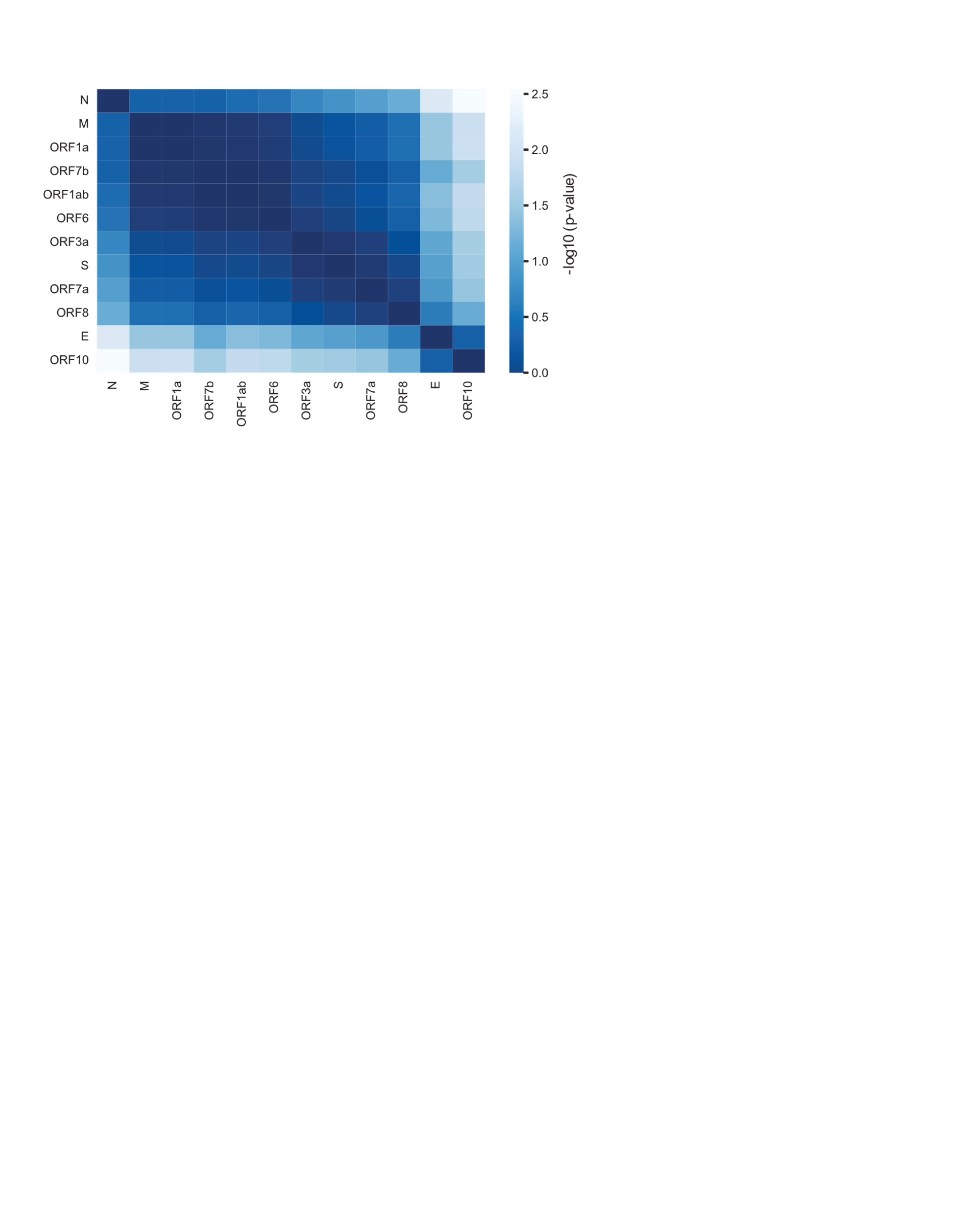


**Appendix Figure S4: Agreement in tAI values between SARS-CoV-2 genes**

A heatmap describing the (-log10) p-values of pairwise T-test that test for significant differences in the tAI values between SARS-CoV-2 genes.

1. Appendix Figure S5


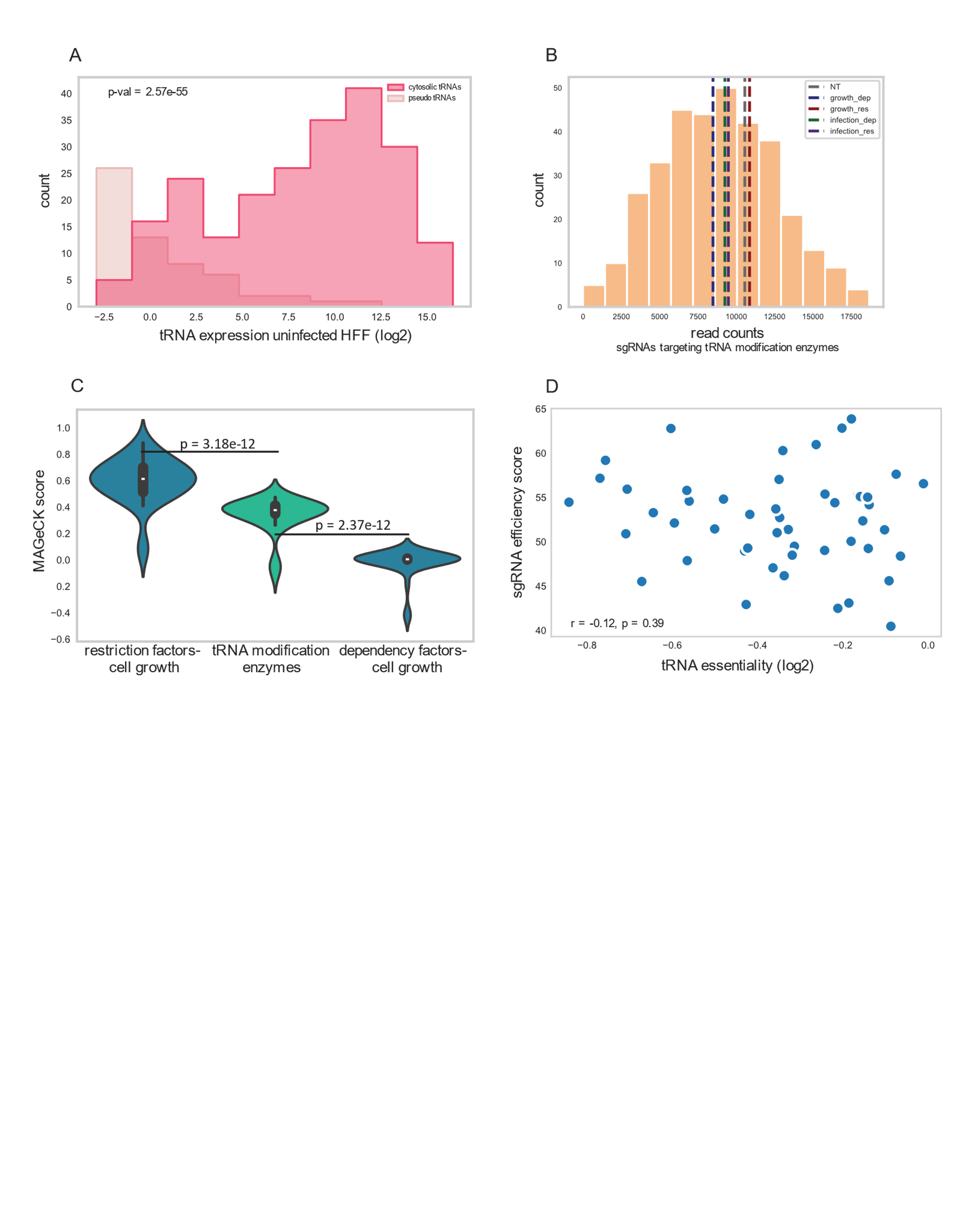


**Appendix Figure S5: Competition experiment using tRNA-CRISPR screen revealed tRNAs that are essential for HFF cell growth**

A| Histograms describing the tRNA levels (log2) of functional cytosolic tRNAs (red) and pseudo tRNAs (beige). The Wilcoxon rank-sum test yielded a statistically significant difference between the distributions (p-value = 2.57e-55).

B| Histogram describing the raw read counts of all sgRNAs targeting tRNA modification enzymes. The dashed lines represent the average read counts of sgRNAs targeting other subgroups: non-targeting (NT, gray), control genes (growth dependency factors, blue), growth restriction factors (red), HCMV-infection dependency factors (green), and HCMV-infection restriction factors (purple).

C| Violin plots comparing the enrichment of sgRNAs in competing cells relative to the ancestor cells taken from (Hein & Weissman, 2022) that target three sub-libraries: restricting factors for cell growth, tRNA modification enzymes, and dependency factors for cell growth. The t-test yielded a significant difference in sgRNA enrichment between sgRNAs targeting tRNA modification enzymes and those targeting the control sub-libraries (restriction factors vs tRNA modification enzymes- p-value = 3.18e-12; dependency factors vs tRNA modification enzymes- p-value = 2.37e-12).

D| Comparison between the tRNA isodecoder essentiality in HFF (x-axis) and the efficiency score of their respective sgRNA, as calculated by the CRISPR sgRNA design tool of Benchling (y-axis). Pearson correlation r = -0.12, p-value = 0.39.

1. Appendix Figure S6


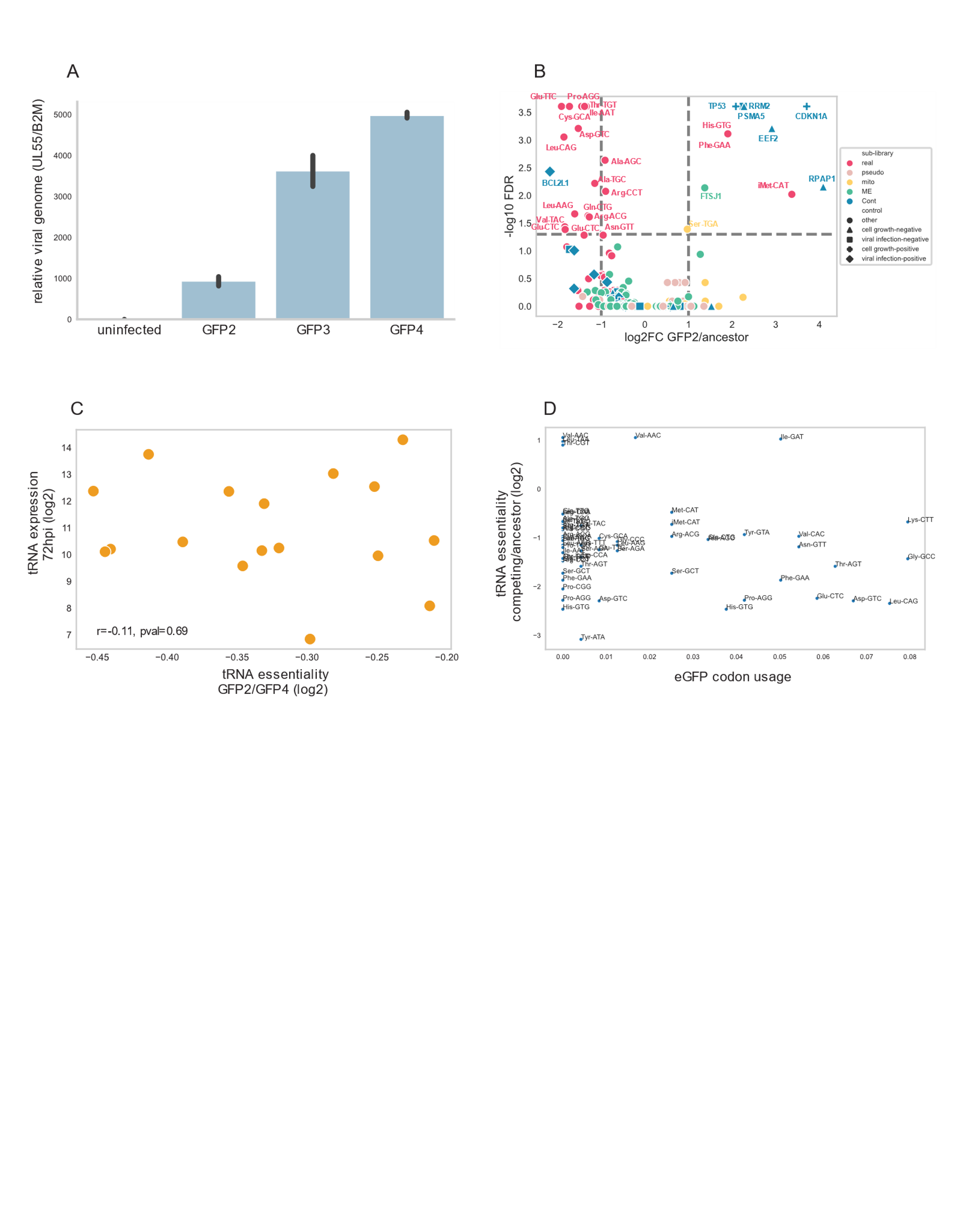


**Appendix Figure S6: tRNA CRISPR screen in HCMV-infected HFF cells identified tRNA genes and modification enzymes that disrupt or improve HCMV infection upon CRISPR targeting**

A| The number of viral genomes estimated by the relative number of UL55 normalized to the B2M human gene, calculated by qPCR, in each GFP-sorted cell population. The error bars depict three technical repeats.

B| A volcano plot for targeted gene hits from tRNA-CRISPR screen in HCMV infection. The x-axis shows the Z-score of log2 FC between lowly-infected cells (GFP2) and the ancestor cell population. The y-axis shows the –log10 FDR as calculated from MAGeCK. The genes are marked according to the sub-libraries. Significance is determined by FDR < 0.05. All values are calculated for three biological repeats.

C| Comparison between the essentiality of the tRNA isodecoder to HCMV infection (x-axis) as determined by the log2 FC of its sgRNA between GFP2 and GFP4 sorted cells and the (log2) expression of the corresponding tRNAs in infected cells, 72hpi (y-axis). Pearson correlation r = -0.11, p-value = 0.69.

D| Comparison between the codon usage of the eGFP gene (x-axis) and the tRNA essentiality for HFF cell growth (y-axis), as determined by the CRISPR screen presented in Fig. 5B.

1. Appendix Figure S7


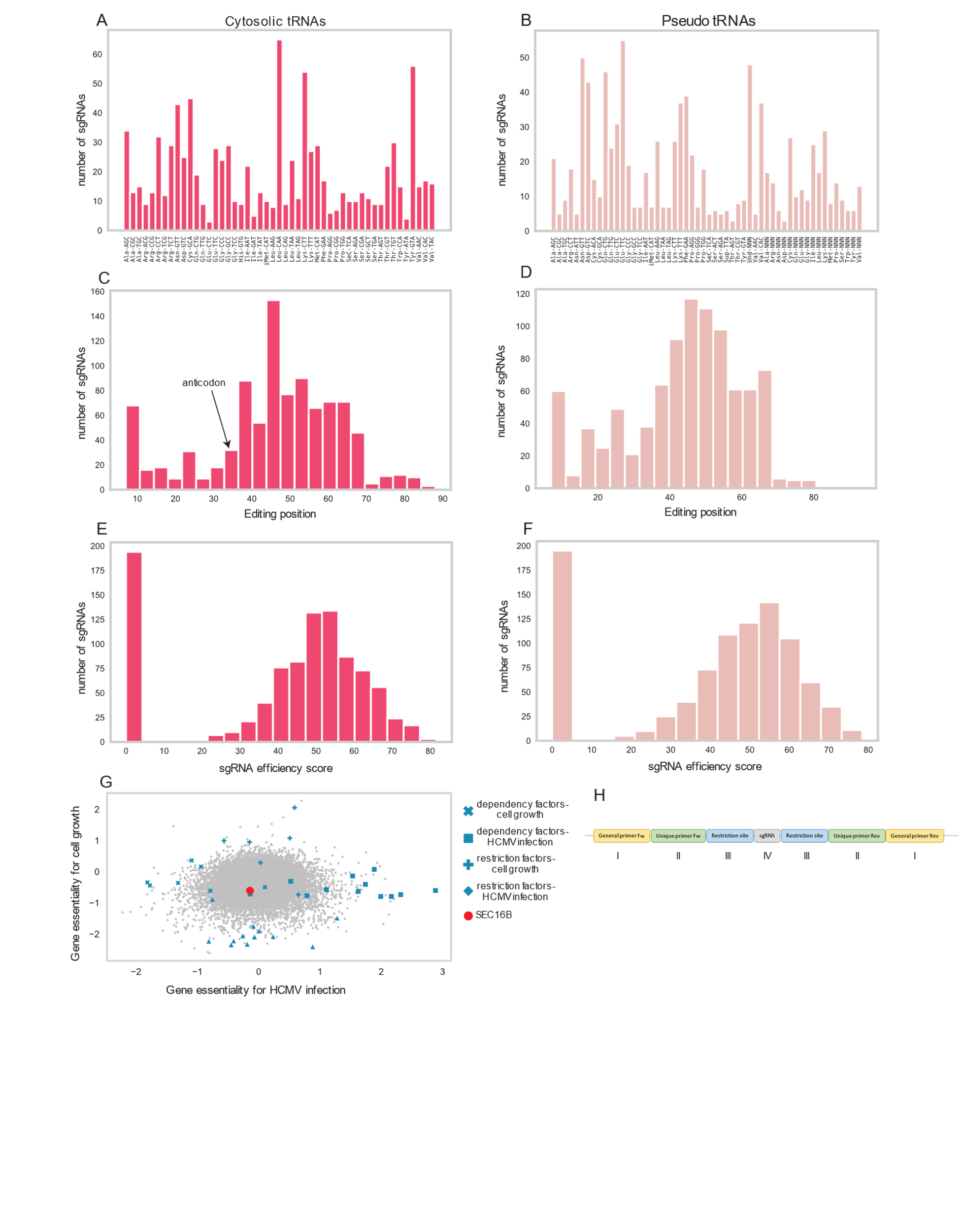


**Appendix Figure S7: sgRNA design parameters**

A-B| Number of sgRNAs (y-axis) targeting each tRNA family (x-axis) of A| functional cytosolic tRNA or B| pseudo-tRNA.

C-D| Histograms describing the editing positions of sgRNAs that target C| functional cytosolic tRNA families D| pseudo-tRNA families. The arrow points to the location of the anticodon in the tRNA gene.

E-F| Histograms describing the sgRNA efficiency score (calculated by the sgRNA design tool of Benchling ([Biology Software] (2022), retrieved from [https://benchling.com](https://benchling.com/))) of sgRNAs targeting E| functional cytosolic tRNA families, F| pseudo-tRNA families.

G| Gene essentiality from a published CRISPR screen of HFF infected with HCMV (Hein & Weissman, 2022). The colored genes are the ones chosen to serve as control genes in the tRNA-CRISPR library, and their marker shape corresponds to the marker described in the legend of Fig. 5B. The restriction factor SEC61B, which did not show the expected effect in the screen, is marked in red.

H| Description of the final oligo design that contains I- General primers (common to all sgRNA variants), II- sub-library specific primers (to allow selective amplification of each sub-library), III- restriction sites for BsmB1 restriction enzyme, IV- unique sgRNA.

1. Appendix Figure S8


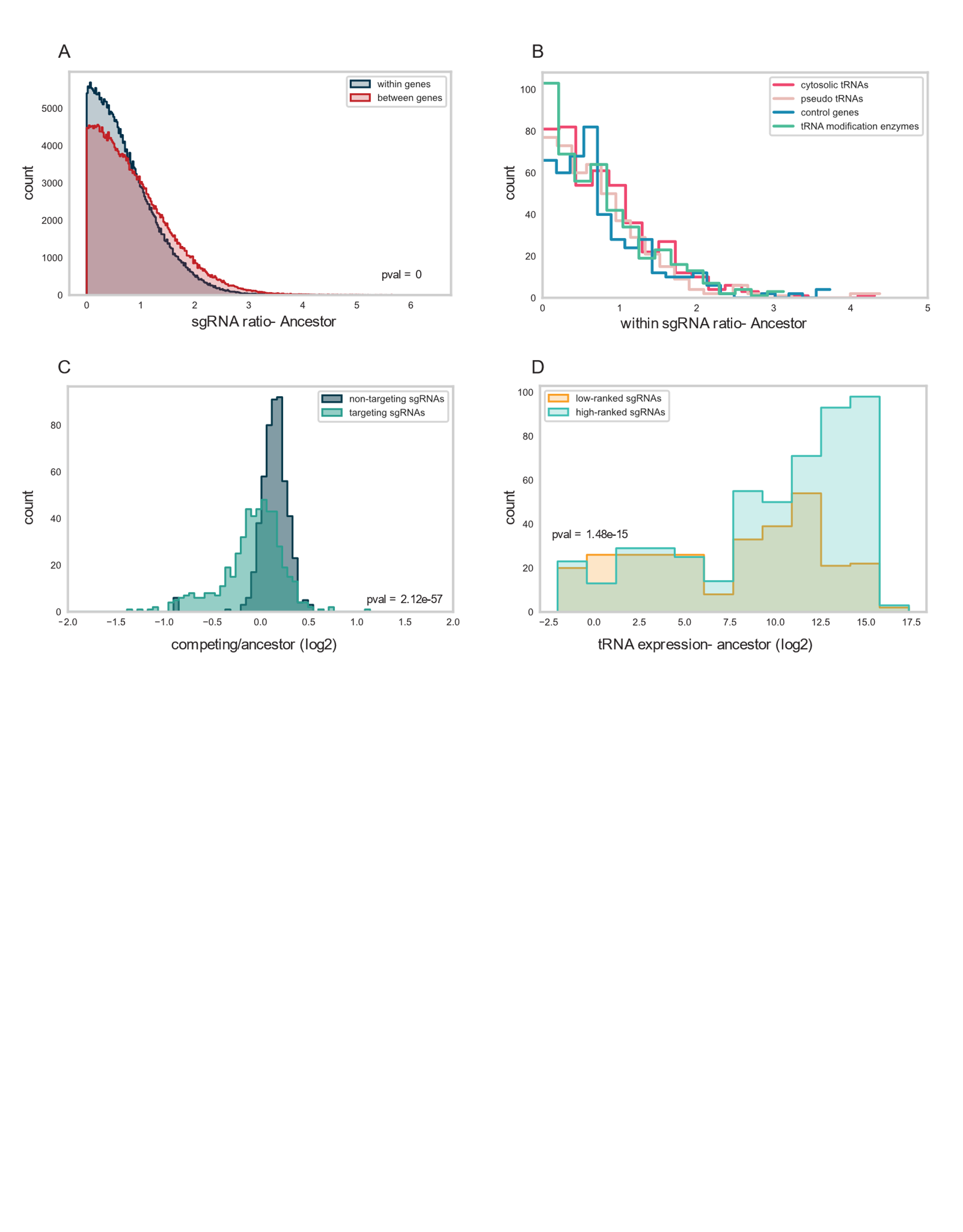


**Appendix Figure S8: sgRNA targeting performance**

A| Histogram of the ratios between normalized read counts of pairs of sgRNAs that target the same gene ("within genes", blue) and pairs of sgRNAs that target different genes ("between genes", red). Data is shown for the ancestor population. Wilcson rank-sum test p-value = 0.

B| Histograms of the ratios between normalized read counts of sgRNAs that target the same gene ("within genes") for different sub-libraries in the ancestor population.

C| Histograms comparing sgRNA enrichment in competing relative to ancestor cells between targeting sgRNAs (light green) and non-targeting sgRNAs (dark green). Wilcson rank-sum test p-value = 2.12e-57

D| Histograms describing the tRNA expression levels (log2) of tRNAs targeted by highly ranked sgRNAs (turquoise) and lowly ranked sgRNAs (orange). Wilcson rank-sum test p-value = 1.48e-15.
